# Remarks on the Treatment of Typhoid Fever by Disulphate of Quinia

**Published:** 1853-02

**Authors:** J. W. Hayward

**Affiliations:** Liverpool, Eng.


*Remarks on the Treatment of Typhoid Fever by Disulphate of Quinia.* By J. W. Hayward, Esq., of Liverpool, Eng.

In our last issue we offered some remarks on the treatment of typhoid fever by large doses of quinia — a subject which, at the present time, appears to be occupying the attention of observers and writers both in this country and in Europe. The efficacy of this mode of treatment is, of course, a matter to be settled by observations made with proper care, reported with candor, and accumulated in sufficient number at different times and places to obviate the liability of attributing to the influence of the remedy, results due to the inherent tendencies of the disease. Thus far, facts pertaining to this subject seem to conflict with each other. We referred in our last number to observations which appeared to show that the quinia does not possess the power to cut short or abridge the duration of typhoid fever. In the Southern Med. and Surg. Journal, we find the following paper, copied from the London Lancet, in which the remedy is stated to have proved apparently highly successful in a large collection of cases. We reproduce the article in our columns in order that it may aid in inciting to further experimental trials of the plan of treatment. It is to be regretted that the report is so concise. More precise statements respecting the symptoms, the duration of cases, etc., would have rendered the paper more satisfactory. The author being unknown, the reliability of the statements must be based on the internal evidence of the article, and of this we leave the reader to judge, without offering any opinion of our own. The article is as follows:

In a practice, private and parochial, of which I have had charge for some time, at the south end of this town, I made particular observations on eighty successive cases of fever of the typhoid type; and I found the first symptom, in twenty of them, was diarrhœa; in twelve, diarrhœa and vomiting; in seven, vomiting alone; the rest began with pain in the head. All had pain in the head afterward; sixty-six describing it as "lightness," fourteen as "heaviness." All had tenderness of abdomen — nine to a great extent. Seventy-one complained of soreness of the flesh over the whole body—some to such an extent that their impression was that they had rheumatism. Seventeen had considerable inflammation of the sub-maxillary glands. Seventy-three had delirium, twenty-one of which were very severe. In all, the tongue became very dry, brown, hard, and cracked; the first crack was generally a deep, longitudinal one down the centre of the tongue, (even whilst it was clammy and velvety in appearance, and of a milk-and-water-color,) extending from the base to nearly the apex; then many transverse and oblique ones. In twenty-four, the skin became rough and brown, with petechiæ observable. In all the thirst was intense; and the other symptoms of fever were not less evident; therefore suffice it to say they were well-marked cases of fever of the typhoid character.

In twenty-seven the treatment was commenced in the "first stage;" in fifty-three in the "second stage." Three were fatal. All the rest recovered more or less quickly.

The principal treatment in all except one was the use of disulphate of quinia, — so much recommended by Dr. Dundas, of this town, — of course modified according to the predominating symptoms. Thus, if I found the pulse quick, weak and thready; the tongue cracked, brown, and dry, (rough or smooth;) great thirst and delirium; no appetite; tenderness of abdomen; soreness of flesh, &c., I put the patient upon disulphate of quinia, in solution, at once; four or five grains every two hours. If great restlessness, and no sleep, I added three or four minims of tincture of opium to each dose. If general sinking of vital powers, some wine or brandy, with beef tea. If the solution of quinia were vomited, it was given in an equal quantity of wine, or wine and water. If the patient continued sinking, I increased the quantity of quinia, but never had occasion to extend beyond seven grains per dose. When ringing in the ears occurred, the quantity was decreased, but still kept up till there was a good appetite. If the delirium was intense, the pain in the head described as heavy, (which were always strong subjects, whose bowels were confined,) with strong pulse, a dose of chloride of mercury, and sometimes a blister to the nape of the neck. If tenderness of abdomen was great, a few leeches or sinapism. If vomiting, a sinapism over the stomach. If diarrhœa, a little calomel and opium, or diacetate of lead and opium; though this symptom sometimes required nothing more than the tincture of opium given with the quinine.

In seventy-nine cases, marked improvement was observable in the course of twelve hours: the pulse was the first to improve, then the delirium to give way, then the pain in the head, the thirst, the soreness of flesh, tenderness of abdomen, dryness of tongue, and the appetite to improve. A pulse of 120, small and thready, would become 90, softer and fuller; and in the majority of the cases the pain in the head and delirium ceased entirely in the same period, and a rapid improvement followed; with the small exception of two cases of children ten and eleven months old, neither of whom had the medicine regularly, and both were complicated with bronchitis. The eightieth case was that of a woman, in which quinia was not used. These three died, evidently from typhus. If the typhoid symptoms were allowed to become well-marked, and the patient to sink much, his recovery was slow, and he required wine or brandy. I feel confident, from personal observation, that were the disulphate of quinia to be used promptly, and to cinchonism, the mortality even of typhus itself would be very small.

P. S. The practice alluded to is that of Dr. Whittle, who wishes me to make this communication to you.

